# Our New York Letter

**Published:** 1887-04

**Authors:** 


### Correspondence.

### OUR NEW YORK LETTER.

YOUNG PHYSICIANS IN NEW YORK—THEIR STRUGGLE FOR A LIVELIHOOD—THE ABUSE OF MEDICAL CHARITY—NEW YORK NOT A GOOD PLACE FOR YOUNG DOCTORS—DR. SIMS' MONUMENT—TRANSPLANTING A RABBIT'S EYE TO A HUMAN SUBJECT.

*To the Editors of the Atlanta Medical and Surgical Journal:*

It is a pretty well known fact that the majority of the younger doctors in New York, and not a few of the older ones also, are obliged to make a hard struggle for an existence. If the facts were known, I believe that quite a fair percentage of the profession depends largely upon other resources than the income of their practices for living expenses. These latter ones would certainly be unable to support themselves as they now live, or to support themselves at all, if depending upon practice for a livelihood. When we consider the greatly over-crowded condition of the medical profession in New York, and the abuse of medical charity, these statements may be easily accounted for. The rivalry between doctors here is certainly very great, and leads in many instances to rather dishonorable methods for obtaining patients. Dispensaries and hospitals are on the increase, and the opinion is growing among the people that it is useless to pay for what can be had for nothing. At many of the dispensaries all applicants are treated without any questioning as to their financial condition. With few exceptions the investigations, if any, are very superficial, and the patients' statements are accepted as the truth. Many of our wealthy physicians, with large private practices, preside over dispensary classes and employ clinical assistants.

They are anxious to have large classes, either through pride or for purposes of special study. One assistant recently told me of an attempted investigation on his part where he was satisfied that the patient could pay for medical treatment, and he accordingly refused to attend her. The patient entered a complaint and the young assistant lost his position thereby. Many of the wealthy people here are now taking advantage of the private rooms at the hospitals, where board and the best medical attendance may be had at prices ranging from $20 to $50 a week, and where the doctor gets nothing but experience. Very little surgery here falls into the hands of the young practitioner. Most of the surgical cases occur among the laboring classes, and the ambulance carries them off to the hospital, where they are welcomed. The wealthier patients can afford to employ surgeons of established reputation, and consequently, however well prepared a young doctor may be to treat broken limbs, even after a thorough hospital training, his chances for showing what he can do will be very slim. In many parts of the city doctors are so numerous that the rivalry leads to a reduction of fees. Doctors living near tenement districts are expected to make visits, including long distances and climbing to the tops of five-story houses, for $1.00. If the doctor refuses, there are always plenty of others in the neighborhood with large practices and good reputation among their patients, who will go for this fee. Occasionally I have had doctors tell me of patients making an office visit and expecting only to pay fifty cents, saying that doctor so-and-so (a good man) only charged half a dollar in the office. The only reply to such remarks is that doctor so-and-so probably knows the value of his services. There are many amusing stories told of the means resorted to sometimes for getting patients. A doctor living on a block where physicians are numerous recently told me the following: A patient of his called at the office one evening and was informed on the stoop by the servant that the doctor was not at home. The patient descended the stoop, and while passing the next house a voice from the window said: "You will find just as good a doctor in here, sir." The man thanked his unseen informer, but said he thought not. Unless there are some special reasons for young physicians locating in New York, they will certainly do better elsewhere.

It will be gratifying to many to hear that progress is being made toward the erection of the monument in honor of Dr. J. Marion Sims. A competition is now being held at the Academy of Medicine to decide upon the selection of a model for this statue. Shortly after the death of Dr. Sims, the *Medical Record* opened a monument subscription list, which resulted in contributions of money from all parts of the country, and at last accounts the sum of $10,000 had been realized. The principal sculptors were then offered an opportunity of submitting their workmanship in the form of models, and at present there are to be seen at the Academy of Medicine two busts and eight standing figures. It is said that some very prominent sculptors considered the sum of money raised very small for a monument and pedestal and declined to enter the contest. Others thought that to produce a figure of Dr. Sims, which could be recognized as life-like, would be too severe a trial of their skill. The entire collection of models is certainly a superior one. That by Wilson MacDonald represents the doctor standing as if delivering a lecture and wearing a Prince Albert coat. The statue of Fitz Green Halleck, in Central Park, was produced by this sculptor. Mr. Turini exhibits three models—one is a standing figure in white plaster with the hands clasped; another is a bronzed bust; the third is a bronzed standing figure holding a note-book and pencil. The work of Mr. Louis Amateis may also be seen in plaster, figure and pedestal. Mr. Alexander Doyle has three models—one is a standing figure with the arm bent and resting on a column, upon which are several books; another is a head of the same size as the author intends the bust in bronze to be; a smaller figure by the same artist is standing, and the hand rests upon a column. Mr. Carl Schmidt has designed a model wearing a fall overcoat and also a bust. All these competing sculptors had the use of the bronze bust of Paul du Bois, and also photographs, as guides from which to copy.

In January, Dr. C. H. May, a clever young physician of this city, began a series of experiments on twenty-four rabbits and acquired considerable skill in the operation of transplanting eyes. Success was obtained in four cases. Dr. May believed that these results justified a trial upon the human subject, and that in case of failure a glass eye could still be used. Patients were rather unwilling to submit to the experiment, as no positive results could be promised; but finally one presented himself. The man was about thirty, with good health, and had lost his right eye through a traumatism when a boy. A Belgian hare was selected with eyes a trifle darker and a very little smaller than the patient's remaining one.

After practicing the operation several times on the cadaver, Dr. May began on the living subject. The rabbit was placed upon a table near the patient and etherized. While ether was being administered to the patient, the right eye of the rabbit was being removed, and all its connections were severed except the optic nerve. The conjunctiva was then cut away from the patient's right eye and black silk sutures passed through either side. The muscles were divided and also held by sutures. The eye now only remained connected by the optic nerve. With a combination forceps, invented by the operator, the nerve was seized close to the eyeball and securely held, while at the same time it was transfixed by a needle passing through it carrying a cat-gut ligature. The eye was then quickly removed. The rabbit's eye was then removed in the same way and placed in the patient's conjunctival sac. This transfer was made in one minute. The two optic nerves were drawn closely together and the sutures tied. The muscles were also drawn together by the silk threads and the conjunctiva was pulled over the eye and sewed to that left surrounding it. Ten stitches were made in all. Both eyes were then closed and vaseline applied to the lids. Cotton compresses were secured by tight bandages and removed three times daily. Hot water applications were freely made to the transplanted eye to aid union. The entire operation consumed one hour and a quarter. A slight corneal haziness appeared on the second day. On the fourth day the silk sutures were removed, and on the sixth day the bandage was taken off. On the evening of the eighth day commencing ulceration of the cornea was discovered, and by the following morning part of the iris had escaped through the corneal perforation. It was then decided to remove the eye and insert a glass one. This was done under cocaine. The muscles, conjunctiva and optic nerve were found firmly united and the cat-gut had been absorbed. A. F.

*New York, March, 1887.*

